# Prenatal Stress Modulates Placental and Fetal Serotonin Levels and Determines Behavior Patterns in Offspring of Mice

**DOI:** 10.3390/ijms252413565

**Published:** 2024-12-18

**Authors:** Victoria Melnikova, Nadezhda Lifantseva, Svetlana Voronova, Nadezhda Bondarenko

**Affiliations:** Laboratory of Comparative Developmental Physiology, Koltzov Institute of Developmental Biology of the Russian Academy of Sciences, 119334 Moscow, Russia; v_melnikova@mail.ru (V.M.); n.lifantseva@gmail.com (N.L.); svetvor@mail.ru (S.V.)

**Keywords:** serotonin, placenta, fetus, prenatal development, stress, behavior

## Abstract

Available evidence from animal studies suggests that placental serotonin plays an important role in proper fetal development and programming by altering brain circuit formation, which later translates into altered abnormal adult behaviors. Several environmental stimuli, including stress and maternal inflammation, affect placental and, hence, fetal serotonin levels and thus may disturb fetal brain development. We investigated the effect of prenatal stress of varying intensities on the formation of adaptive behaviors in mouse offspring and the role of placental serotonin in these processes. Mild prenatal stress increased placental serotonin synthesis, whereas exposure to moderate stress decreased it. Prenatal stress of varying intensities also resulted in multidirectional changes in animal behavior in progeny, consistent with changes in serotonin levels in the placenta and fetal tissues. Mice exposed to mild prenatal stress showed higher sociality and exploratory activity, whereas, after moderate stress, in contrast, they avoided contact with other individuals of their species and had reduced exploratory activity, with no effect on locomotor activity. Thus, in mice, stressors of varying intensities during the critical period of intrauterine development can affect the synthesis of serotonin by the placenta and lead to multidirectional changes in animal behavior in postnatal life.

## 1. Introduction

It is known that in mice the placenta can synthesize serotonin and thus regulate its levels in the blood and tissues of developing fetuses [1,2,3]. Placental serotonin plays an important role in maintaining the required serotonin levels in the fetal brain during the period from day 11 to day 16 of embryonic development (E11–E16) [1]. This is crucial for the regulation of neurogenesis and axonal growth in the brain [4,5]; therefore, any fluctuations in these levels during critical periods of prenatal development can affect the formation of neural networks in the brain [6,7,8] and, hence, the behaviors and adaptations of the animals during their postnatal lives.

Placental serotonin synthesis can be influenced by external stimuli. According to the research literature, moderate stress, inflammation, and exposure to unfavorable factors change the levels of serotonin in the placenta and indirectly affect the formation of innervation of the fetal forebrain [2,4]. Moreover, these changes persist throughout postnatal life [9,10] and may underlie further changes in animal behavior in adulthood. Our recent study demonstrates that increased placental serotonin levels have long-lasting consequences, namely, a reduction in the adrenal medulla size and decreased aggressiveness in male offspring [11].

In this study, we focused on a more detailed investigation of the effects of prenatal stress of varying intensities on the formation of adaptive behaviors in mouse offspring and the role of placental serotonin in these processes. We assessed the development of coping strategies, particularly motor activity, orienting behavior, exploratory behavior and anxiety, and emotional–behavioral reactivity, in new, potentially stressful conditions using standard Open Field and Elevated Plus Maze Tests and social behavior and aggressiveness using the Three-Chambered Social Approach Task and Resident–Intruder Test in male offspring on day 30 of postnatal development (P30).

## 2. Results

### 2.1. Serotonin in the Placenta and Fetal Tissues

The amount of serotonin was determined at E15, immediately after the end of stress.

In the control animals, the total serotonin content in the placenta significantly exceeded that in the fetal brain (2055.49 pmol vs. 47.34 pmol per organ), suggesting that the placenta is the main source of serotonin at this developmental stage.

**Figure 1:**
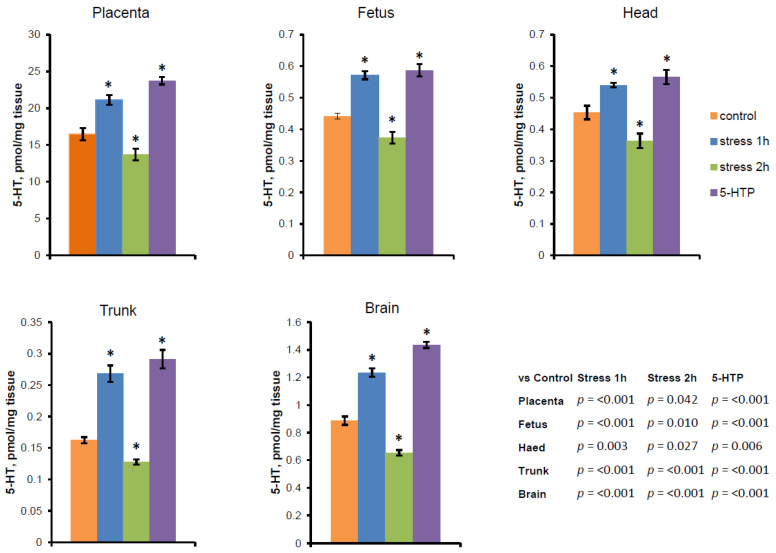
Changes in serotonin concentrations in the placenta and fetal tissues of mice at E15 (in pmol per mg tissue) under the influence of stress of varying intensities and after the administration of serotonin precursor 5-HTP. 5-HT was measured by the HPLC method in placentas, whole fetuses, and fetal trunks, heads, and brains. The data are presented as mean ± SEM, *n* = 9, for each group. * *p* < 0.05 vs. control group. 5-HT—Serotonin, 5-HTP—5-hydroxytryptophan.

Mild stress for 1 h significantly increased while moderate stress for 2 h decreased placental serotonin levels (Figure 1). The serotonin concentrations in whole fetuses, heads, trunks, and brains changed similarly to those in the placenta. We compared the effect of mild stress (1 h) and the introduction of serotonin precursor 5-hydroxytryptophan (5-HTP). The results demonstrated that mild stress (1 h) provided the same increase in serotonin levels as 1 mg/kg of body weight of serotonin precursor (Figure 1).

### 2.2. Behavioral Tests Results

**Figure 2:**
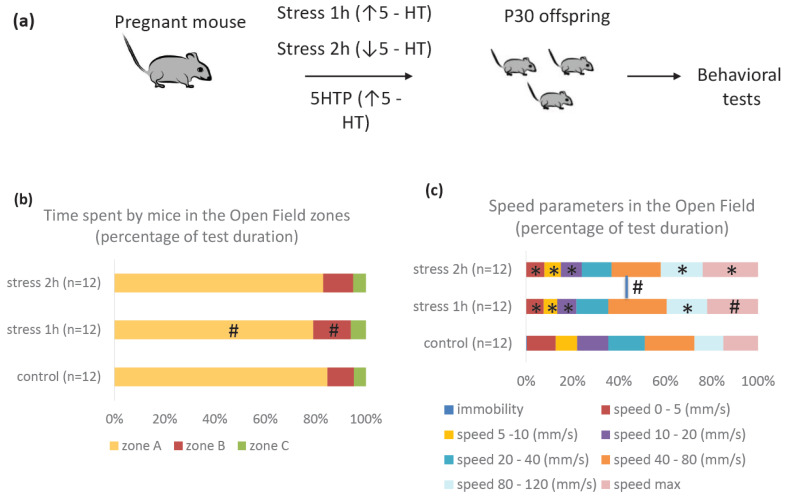

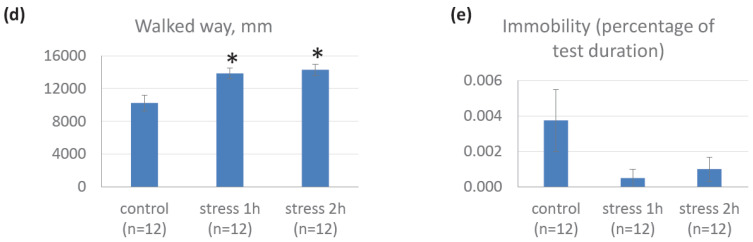
Experimental design (**a**) and behavioral parameters of mice (P30) in the Open Field Test after exposure to stress of varying intensities during the period E11-E14 (**b**–**e**). *n* = 12 for each group, * *p* < 0.05 vs. control group, # *p* < 0.1 vs. control group.

We studied the behavior of mice that experienced prenatal stress of varying intensities during the period E11–E14 at the age of 1 month (Figure 2a). To assess the formation of social interaction strategies, we used the Resident–Intruder Test and the Three-Chambered Social Approach Task. To assess reactions to new conditions, the Open Field and Elevated Plus Maze Tests were used.

#### 2.2.1. Open Field Test Results

The results of the Open Field Test showed that all animals were normally mobile, without abnormalities, and preferred to be in the near-wall zone of the arena (zone A, Figure 2b). After prenatal exposure to mild stress, mice showed a tendency to more actively explore the middle zone of the arena (zone B, Figure 2b) due to less time spent in zone A. After moderate prenatal stress, mice did not differ from the controls in this parameter. There were no differences in the time spent in the central zone (zone C) of the arena.

The control group demonstrated the maximum immobility time in 5 min (0.38% of the observation time) (Figure 2c,e). Since the proportion of immobility in the total motor activity of the animals during the observation period was very small, we placed these values on a separate panel (Figure 2e). However, these differences were not statistically significant. The control animals moved at a speed of up to 20 mm/sec 35.7% of the time, i.e., a rather slow third of the entire observation time. After prenatal stress of varying intensities, the animals had much lower proportions of minimal movement speeds (22% and 24.2%, respectively), i.e., they moved faster than the controls around the arena. The proportions of average speeds were approximately the same in all groups. In the group of animals after prenatal exposure to mild stress, speeds from 40 to 80 mm/sec prevailed, and, in this parameter, they also differed from the animals exposed to moderate prenatal stress, which did not differ from the controls. Mice from both experimental groups moved around the arena by running more than those in the control group. Animals exposed to moderate prenatal stress demonstrated the maximum number of sudden changes in speed: 24.1% of the observation time (Figure 2c, speed max). Thus, we can say the following about some differences in the uniformity of movement between the two experimental groups in the Open Field Test: mice with prenatal exposure to mild stress moved more uniformly at a relatively high speed, while mice exposed to moderate prenatal stress moved more unevenly. The walked way of the animals from both experimental groups was also significantly greater than in the control (Figure 2d).

#### 2.2.2. Elevated Plus Maze Test Results

In the Elevated Plus Maze Test, the animals did not differ in mobility (the speed indicators were not the key parameters for this test, and no behavioral features were revealed). The walked way in both experimental groups also did not differ from the control. However, mice exposed to mild prenatal stress tended to show a decrease in this parameter, while those exposed to moderate stress tended to show an increase (when comparing the two experimental groups, *p* = 0.069).

**Figure 3:**
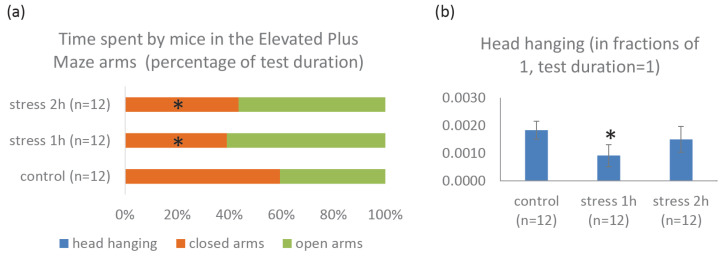
Behavioral parameters of mice (P30) in the Elevated Plus Maze Test after exposure to stress of varying intensities during the period E11–E14. (**a**) Time spent by mice in the Elevated Plus Maze arms, (**b**) Head hanging. *n* = 12 for each group, * *p* < 0.05 vs. control group.

Mice after prenatal exposure to mild stress were significantly less likely to hang their heads down from the open arms of the maze than the control mice, while mice after moderate stress did not differ from the controls (Figure 3b). The animals from both experimental groups spent significantly less time in the closed arms than the controls due to an increase in time spent in the open arms of the maze, respectively (Figure 3a).

#### 2.2.3. Resident–Intruder Test Results

**Figure 4:**
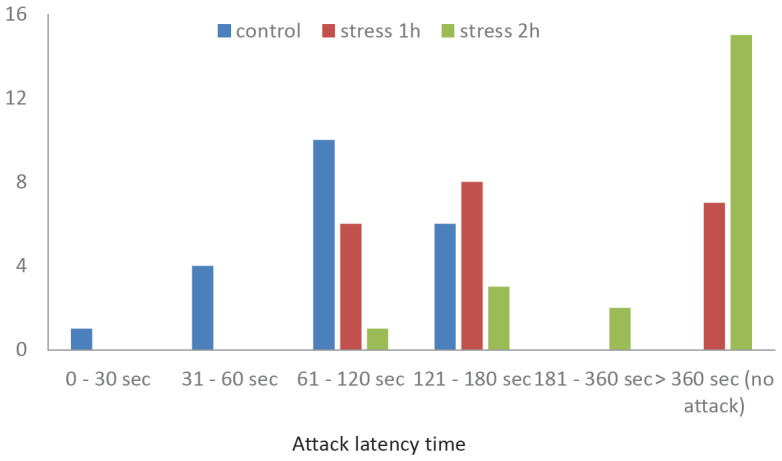
Effect of prenatal stress of varying intensities on the propensity to defend one’s territory in mouse offspring (P30), expressed as the number of intruder attacks, according to the Resident–Intruder Test. *n* = 21 for each group.

All animals in the control group attacked the intruder within 20–172 s, i.e., all attacks occurred within 3 min of interaction (Figure 4); 15 animals out of 21 attacked within the first 2 min. After prenatal exposure to mild stress, no resident attacked the intruder within the 1st minute; within the 2nd minute, 6 out of 21 animals attacked. In this group, there were also 7 animals that did not attack the intruder during the entire 6 min of observation.

After moderate prenatal stress, only 6 out of 21 animals attacked the intruder within 6 min, while two attacks were non-standard (without a squeak or weakly expressed). In the attacking animals, the latent period before the attack was 72–192 s (from 123 s onwards for “standard” attacks), and only 1 mouse out of 6 attacked after less than 2 min of interaction.

#### 2.2.4. Three-Chambered Social Approach Task Results

**Figure 5:**
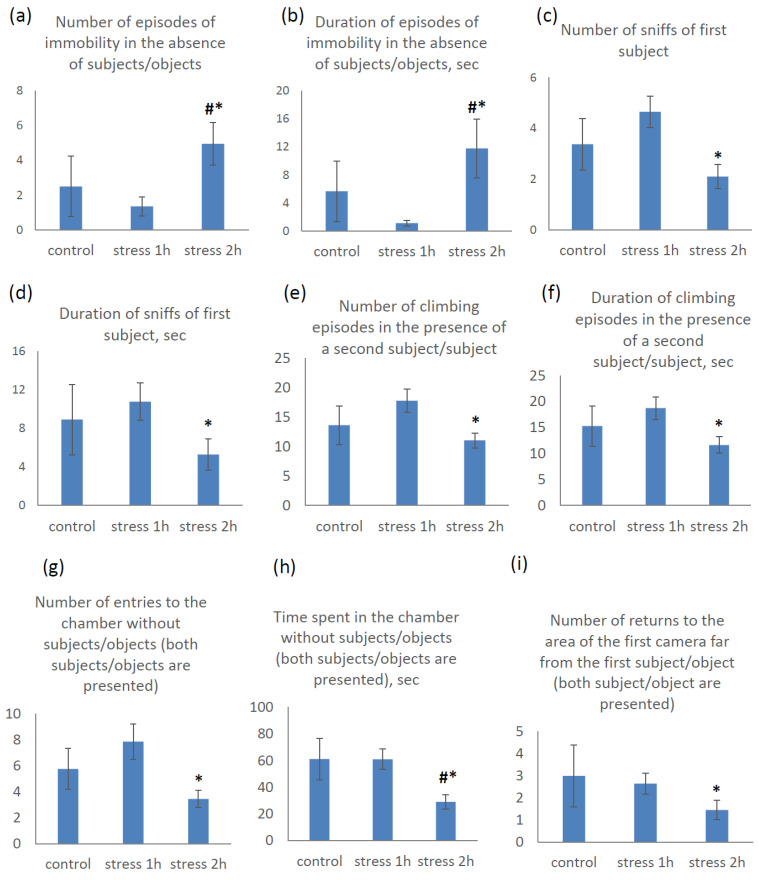
Behavioral parameters of mice (P30) in the Three-Chambered Social Approach Task after exposure to stress of varying intensities during the period E11–E14. *n* = 8 for control group, *n* = 20 for both experimental groups. * *p* < 0.05 vs. 1 h stress group, **#** *p* < 0.1 vs. control group.

When the animal was placed under the initial conditions of this test in the absence of subjects/objects (the first 5 min, both doors to the adjacent chambers were closed), we obtained differences in the number and duration of episodes of immobility (Figure 5a,b). Prenatal exposure to mild stress led to a decrease in immobility (the animals actively walked) and moderate stress led to an increase in this parameter. If, in the case of mild stress, these differences were not statistically significant, then, when comparing the group of animals exposed to moderate stress with the control, both cases represented a trend (*p* = 0.09, #), and the differences between the experimental groups were statistically significant (*p* < 0.05, *), which indicated the opposite directions of the effects of these two experimental influences.

After the presentation of the first subject and object (in the second 5 min, free passage to one chamber with a mouse in a cylinder), we obtained clear differences in the numbers and durations of mutual sniffing encounters of the mice («nose-to-nose») (Figure 5c,d). Prenatal exposure to mild stress again led to a slight increase in the numbers and durations of these direct contacts, while prenatal exposure to moderate stress led to a decrease in these parameters. Moreover, statistically significant differences were again revealed between the two experimental groups (*p* = 0.003 and *p* = 0.015, respectively). Thus, mice with prenatal exposure to mild stress were more interested in the new subject, while those exposed to moderate stress, on the contrary, showed much less interest.

With the possibility of free passage between all chambers (the third 5 min, both doors open, free access to both subjects and objects), we obtained pronounced differences in several parameters (Figure 5e–i). At the same time, the behavior in the presence of the second subject/object was somewhat different than that in the presence of the first subject/object only, since this situation was no longer fundamentally new for the animals. In this case, we observed approximately the same effects of prenatal stress of varying intensities as in the previous cases: prenatal exposure to mild stress led to a slight increase in the number and duration of climbing episodes on the rods of the cylinder or the walls of the chamber, while prenatal exposure to moderate stress led to a slight decrease. Significant differences were also observed only between the two experimental groups (*p* = 0.015 and *p* = 0.022, respectively).

Also, when allowed to move freely between all experimental zones, mice after prenatal exposure to mild stress went to the chamber without subjects/objects (central) more often. At the same time, the amount of time spent in this chamber did not differ from the control values. Mice after prenatal exposure to moderate stress went to this zone less often and spent less time in it. In both cases, the differences between the two experimental groups were reliable (*p* = 0.004 and *p* = 0.003, respectively); in the case of the duration of stay in this zone, there was a pronounced tendency towards its decrease after prenatal exposure to moderate stress compared with the control (*p* = 0.063).

Another additional indicator of a decrease in exploratory activity in mice with prenatal exposure to moderate stress was the number of returns to the chamber with the first subject/object when given the opportunity to move freely between all chambers. The control animals were quite heterogeneous in this parameter. Significant differences were again found between the two experimental groups (*p* = 0.035). It is worth noting that in these cases, in almost all observations, significant differences were revealed between the two experimental groups. In other words, prenatal exposure to stress of varying intensities led to multidirectional changes in mice behavior relative to the controls.

## 3. Discussion

### 3.1. Stress-Induced Modulation of Serotonin Levels in Placental and Fetal Tissues

According to the research literature, the placenta is a significant source of serotonin in mice. It is known that maternal serotonin cannot pass through the placenta to the fetus [1]. The peak activity of the placenta as a source of serotonin in mice occurs during the period E11–E16. Thereafter, the synthesis of serotonin in the fetus’s own tissues increases, and the role of the placenta becomes less significant [1]. Our data show that the total serotonin content in the placenta at E15 is two orders of magnitude higher than that in the fetal brain. Thus, we can assume that we exposed the animals to experimental influences at the developmental stages, when the placenta plays a leading role in maintaining the levels of serotonin necessary for proper fetal development. We have shown that stress affects the levels of serotonin in the placenta, which is generally consistent with the research literature data [4,12,13,14]. However, the direction and magnitude of these changes may depend on the intensity of the exposure. According to our data, stress of varying intensities had a multidirectional effect on the serotonin levels in the placenta and fetal tissues. Mild stress during the studied period of development increased and moderate stress decreased the levels of serotonin. Analysis of the literature data indicates that stress susceptibility depends on the strain of the mice. While, according to our data, in BalbC mice, moderate stress reduced the levels of serotonin in the placenta, in C57/Bl6 mice, the same stress had the opposite effect [12].

It can also be assumed that the effect of stress may depend on the stage of development. The mechanisms of such changes have not yet been studied in detail, but there are some data that associate this with changes in the concentration of free tryptophan in the mother’s blood, the availability of 5-HTP, and the activity of tryptophan hydroxylase [2,4]. A review by St-Pierre et al. [13] discusses the effect of prenatal maternal stress on the glucocorticoid (11β-Hydroxysteroid-Dehydrogenase-2, glucocorticoid receptors, and corticotropin-releasing hormone) and serotonin (SERT, serotonin receptors) systems, as well as the interaction of serotonin and glucocorticoids in the placenta. For example, the methylation of placental HTR2A (the gene encoding the 5-HT2A receptor) can influence placental growth and function and placental serotonin signaling, which, in turn, can influence fetal brain development, causing long-term neurobehavioral consequences [15,16,17]. The gene encoding SERT, SLC6A4, is also subject to epigenetic regulation [13,18,19]. Chen et al. [12] discuss the mechanism of regulation of placental tryptophan availability and serotonin synthesis mediated by cytokines, particularly by increased levels of the CCL2 chemokine, and the placental response to maternal stress was thus similar to that to inflammation.

We also found changes in serotonin levels in fetal tissues as a result of our experiments similar to those in the placenta (Figure 1). According to the literature, changes in serotonin levels in the placenta after stress may have consequences for fetal brain development [10]. Both too-low and too-high levels of serotonin in the fetal brain may be important for the formation of interneuron connections [10] since serotonin receptors in rodents appear quite early in ontogenesis, even before the formation of these connections, and serotonin can have a morphogenetic effect on these processes [20,21]. In mice, axonal growth actively occurs during the E11–E16 period [1], when the placenta functions as the leading source of serotonin. Thus, changes in serotonin levels in the brains of animals during critical periods of intrauterine development may lead to changes in the formation of interneuron connections and, consequently, behavior in adulthood. These behavioral alterations may to some extent influence the ability of animals to respond to changing environmental conditions, which may be important for the survival of the population.

### 3.2. Long-Lasting Consequences of Prenatal Stress-Induced Modulation of Placental Serotonin Levels

Our behavioral tests assessed the animals’ ability to navigate in new, potentially stressful conditions and their social interaction, which is an important aspect of rodent behavior. This study was carried out in males, in line with published data showing that in females whose mothers were exposed to stress during pregnancy, subsequent changes in behavior differed from those in males and often affected aspects such as anxiety and depression. In males, the changes mainly concerned aspects of social behavior [8]. The tests we used characterize changes in social behavior that are characteristic of males. It is also known that male offspring are more sensitive to prenatal stress than female offspring [14].

The Open Field Test is generally used, especially in pharmacology, to assess the general level of motor activity in animals, which may reflect exploratory activity, immobility, and other aspects, as well as anxiety, although this is quite controversial in the literature [22,23]. The greater the anxiety, the less willing an animal is to explore a new environment. In our case, we considered mainly motor activity and the duration of episodes of exploring the central zones of the arena, which reflected the animals’ activity in general, including exploratory activity.

The results showed that mice exposed to mild prenatal stress spent less time in areas close to a wall (zone A) than other groups (Figure 2a, zone A) and more time in the adjacent middle area (zone B). This may indicate that these animals were less anxious in the new environment and therefore more active in exploring the arena. Animals with moderate prenatal stress, although moving much more than the controls, did so mainly in zone A. Thus, we can see that animals with prenatal exposures capable of influencing serotonin levels both in the placenta and in the fetus differed from the controls in some behavioral parameters at 1 month of age. In this case, some parameters in the two experimental groups looked the same in numerical terms, while others, on the contrary, indicated differences in their behavior. For example, the proportions of low speeds decreased in both experimental groups, which may indicate that they were less cautious. However, in terms of staying in different areas of the arena, this may rather suggest that mice with mild stress may have had lower anxiety and greater exploratory activity, while mice with moderate prenatal stress may have had lower attention and increased activity as compensation. A clearer understanding of this phenomenon requires more specialized behavioral tests of attention and learning.

The effects of changes in fetal serotonin levels on the behavior of the same animals in adulthood, particularly locomotor activity, are quite often discussed in the research literature. Studies, for example, of SSRIs have addressed this [24,25,26]. The accumulated evidence contains many different study designs, with a number of them showing no clear correlation between long-lasting behavioral effects and changes in fetal serotonin levels. However, one study showed that the administration of fluoxetine to rats from E11 onwards before birth resulted in increased anxiety in adult offspring in the Elevated Plus Maze Test. The social behavior of these rats was also altered: play behavior and social contact were reduced and self-care increased [27]. Another study using intraperitoneal injections of fluoxetine in mice between E8 and E18 showed that anxiety also increased in the offspring [28]. However, changes in fetal serotonin levels may influence offspring behavior, particularly anxiety, locomotor activity, and social interactions.

The results of the behavioral assessment of mice in the Elevated Plus Maze Test generally correlated with those for the Open Field Test. Since this test is more relevant for assessing animal anxiety than the Open Field Test, it can be said that animals from both experimental groups showed less anxiety.

In rodent populations, social interactions are very important, so we investigated this aspect in two tests: the Resident–Intruder Test, which allowed us to assess aggressiveness and coping strategies, and the Three-Chambered Social Approach Task, the results of which indicated the animals’ interest in new subjects (exploratory activity) and objects (social interest, anxiety) [29]. The influence of serotonin during prenatal development on social interactions in adult animals and humans has also been extensively discussed in a number of papers [30,31,32,33].

According to the results of the Resident–Intruder Test, animals from both experimental groups differed from the controls. After prenatal exposure to mild stress, the mice showed much greater social flexibility and lability and low aggressiveness, whereas the control mice were characterized by a desire to protect their territory. After moderate prenatal stress, the animals also showed less aggression; however, this behavior looked slightly different: these animals were more likely to avoid the intruder, whereas, after mild stress, they were actively interested in the intruder but attacked less.

We used the Three-Chamber Social Approach Task to determine how mice, following prenatal stress of varying intensities, behaved towards conspecifics that did not pose a potential threat to them: interacting on neutral territory and avoiding the possibility of a direct attack.

Thus, under baseline conditions, when mice did not see other individuals or new objects in their environment, prenatal exposure to mild stress resulted in an increase in mobility and moderate stress resulted in its decrease. When the same animals were tested in the Open Field Test, we observed similar increases in mobility in both groups compared to the controls, although we did not obtain significant differences. In this case, it can be assumed that factors such as smells or sounds made by other animals in the vicinity may have played an important role. That is, the behavior of the animals in the two experimental groups changed in a predominantly social manner. The presence of other mice in the vicinity may not have affected the mobility and activity of mice with prenatal exposure to mild stress but may have led to reduced locomotor activity and, possibly, exploratory activity in animals with exposure to moderate prenatal stress. Furthermore, here, we observed multidirectional effects of prenatal exposure to stress of varying intensities on this behavioral parameter, which was consistent with the multidirectional changes in the levels of serotonin in the placenta and fetuses after these experimental exposures. Similar multidirectional changes were found for the number and duration of direct contacts («nose-to-nose» sniffing) with the first presented animal in the cylinder. Mice with mild prenatal stress were more interested in a new subject, while those with moderate stress, on the contrary, showed much less interest. Since «nose-to-nose» sniffing is one of the first and most important behavioral acts in rodents when meeting a new individual, these data confirm our observations obtained in the Resident–Intruder Test.

In a situation where the animal was able to move freely around all zones and approach any objects and subjects, we obtained the most pronounced differences in such parameters as climbing with support on the walls of the chamber or the rods of the cylinder, the frequency and duration of the stay in the chamber without subjects/objects, and returns to the chamber with the first presented animal. Climbing is generally not a socially significant parameter, but it may reflect the motivational component of exploratory behavior. The animal explored more actively or did not explore the chamber (support on the walls), the object, or the subject (support on the rods of the cylinder). Therefore, it can be assumed that after prenatal exposure to mild stress, mice demonstrated slightly more active exploratory behavior in a relatively familiar environment, while, after moderate stress, they demonstrated less active behavior. Also, after prenatal exposure to mild stress, mice more often entered the chamber where there were no subjects/objects (central), which may also indicate higher motor and exploratory activity. After prenatal exposure to moderate stress, mice entered this zone less often and spent less time in it, which may indicate less active movements throughout the experimental zone. Another additional indicator in favor of a decrease in exploratory activity in mice with prenatal exposure to moderate stress was the number of returns to the chamber with the first subject/object when given the full freedom to move between all chambers. Therefore, it can be assumed that after prenatal exposure to mild stress, mice demonstrated slightly more active exploratory behavior in a relatively familiar environment, while, after moderate stress, they demonstrated less active behavior. Also, after prenatal exposure to mild stress, mice demonstrated higher motor and exploratory activity in contrast to moderate stress. That is, we saw differences in the changes in parameters related more to exploratory activity in general than to social interactions only by the end of the experiment when the animals had already oriented themselves in the surrounding space. Presumably, this was due to a decrease in motivation or attention in mice exposed to moderate prenatal stress. This reaction may also have been secondary because of the so-called stress from the previous social interaction with the first mouse. However, this issue requires further research.

It is interesting and important to note that in these cases, significant differences between the two experimental groups were found in almost all observations. Prenatal exposure to stress of varying intensities led to stable multidirectional changes in behavior relative to the controls. These changes corresponded to those in the levels of serotonin in the placenta and fetal tissues at E15. Thus, prenatal exposures that can lead to changes in the serotonin levels in the placenta can also influence the behavior of animals in postnatal life. The effects of mild stress and moderate stress during a certain critical period of intrauterine development can lead to the formation of two types of adaptive behavior: some animals demonstrated higher sociality and exploratory activity, while others, on the contrary, avoided contact with other individuals and had reduced exploratory activity. At the same time, in both experimental groups, motor activity (without social stress) increased and aggressiveness decreased.

Possible mechanisms of these phenomena are discussed in detail in an article by Kameneva and Melnikova [11]. Changes in serotonin levels in fetal tissues in rodents lead to changes in the development of the adrenal glands (smaller size, medulla). The adrenal glands themselves are not only part of the monoamine system but also a powerful endocrine organ that is sensitive to serotonin levels in prenatal ontogenesis [11,34]. They are responsible for the secretion of stress hormones, so any endocrine influences during their formation may have consequences with respect to an animal’s stress response in postnatal life, upon which the formation of behavior patterns in different life conditions may depend.

It should be noted that the mechanisms discussed may be caused not only by the peripheral but also by the central compartment of the hypothalamo–pituitary–adrenal axis. Of particular interest is the stress-induced modulation of hypothalamic development since this brain region is involved in regulating not only hormone secretion and metabolism but also social and defensive behavior. This is the most affected behavioral aspect in the offspring. During development, serotonin in the brain is involved in a variety of neurodevelopmental processes, such as neuronal proliferation and migration as well as initial axon targeting and circuitry maturation [14]. Animal and clinical studies demonstrate that maternal stress affects the serotonergic system, neurodevelopment, and behavior in offspring [14]. The effects of maternal stress on the placental and fetal serotonin systems are often discussed as a potential underlying factor for excessive fetal cortisol levels. Alterations in fetal serotonin may increase the risk of the onset of neuropsychiatric disorders, such as autism, depression, and anxiety. Human studies clearly show that prenatal stress affects neurodevelopment, including functional and structural brain connectivity alterations involving the amygdala and frontal cortex and changes in hypothalamo–pituitary–adrenal axis functionality. In turn, these alterations induce long-lasting effects on children’s mental health [35]. Studies concerning the role of maternal and fetal serotonin levels in relation to stress, however, are scarce.

The presence of different behavioral patterns in a population of animals, especially those that are social and living in groups, may have important ecological significance. For example, stress can occur in pregnant females when environmental conditions change, and this will have consequences for the offspring, depending on the stress intensity. Studies focusing on wild rodents have shown that elevated stress levels in pregnant mothers, associated with a strong increase in population density, increase serotonin levels in the placenta and embryo. This, in turn, leads to a decrease in chromaffin organs (adrenal medulla) in the offspring, alters hormonal profiles, and promotes migration and settlement in new areals [11]. In our experiments, prenatal exposure to 5-HTP, as a mild stressor, also increased serotonin levels in the placenta and fetal tissues and led to more flexible social behavior in postnatal life. After moderate prenatal stress, animals became more «asocial» and probably less attentive, which may affect their survival in changing environmental conditions. The ratio in a population of the numbers of animals with different types of behavior, especially in species with pronounced social interaction, generally determines the population’s ability to survive both in stable and changing environmental conditions. For example, an increase in the proportion of animals demonstrating reactive behavior within a group may give this group an advantage in unstable environmental conditions and play a role in the survival of the population.

Thus, in mice, stressors of varying intensities during critical periods of intrauterine development can affect serotonin synthesis by the placenta and lead to multidirectional changes in serotonin levels in this organ and, consequently, the whole developing organism, which, in turn, can lead to multidirectional changes in animal behavior in postnatal life.

## 4. Materials and Methods

### 4.1. Animals and Experimental Design

This work was carried out on BalbC mice. The animals were kept in a specialized vivarium, in which they were provided with free access to water and food, ventilation, and controlled lighting. All procedures with animals that could cause them pain or other painful conditions, including euthanasia, were performed under general inhalation anesthesia with isoflurane (SomnoSuit rodent anesthesia device, Kent Scientific, Torrington, CT, USA).

To obtain mice with dated pregnancy, four females were placed in a cage with a male in the evening, and the presence of a vaginal plug was visually analyzed in the morning. Pregnant females were subjected to stress of varying intensities (immobilization for 1 h or 2 h) daily during the period E11–E14. The mice were immobilized using a 50 mL conical tube with perforations to allow for ventilation [12], while the nonstressed group was left undisturbed. During the same period, another group of pregnant females received the serotonin precursor 5-HTP orally daily at a dose of 1 mg/kg. It has previously been shown that oral administration of the solution to mice does not affect serotonin levels in the placenta [11]; thus, intact mice served as a control.

In stage E15 (*n* = 9 for each group), the placentas and fetal tissues were quickly dissected on ice, homogenized with an ultrasonic homogenizer (BandelinSonopuls, Burladingen, Germany) at 4 °C in 0.1 M HClO4, and centrifuged at 10,000× *g* for 20 min at 4 °C. The supernatants were collected and stored at −80 °C prior to measurements of serotonin content by HPLC.

Male offspring from mice exposed to stress were raised to postnatal day 30 and subjected to behavior tests: Resident–Intruder Test, Open Field Test, Elevated Plus Maze Test, and Three-Chambered Social Approach Task. For the Open Field Test, Elevated Plus Maze Test, and Three-Chambered Social Approach Task, we used equipment for analyzing the behavior of small rodents (“Open Science”, Russia), with video recordings of behavioral experiments and computer analysis using a specialized software package with neural network behavior recognition.

### 4.2. Serotonin Analysis by HPLC-FLD

An Agilent 1260 Infinity II High Performance Liquid Chromatography with Fluorescent Detection (HPLC-FLD) system (Agilent Technologies Inc., Waldbronn, Germany) was used for the serotonin analysis. Analytes were separated using a reverse-phase InfinityLab Poroshell 120 EC-C18100 mm × 4.6 mm column with a 2.7 μm particle size (Agilent Technologies Inc., Waldbronn, Germany). The column was thermostated at 30 °C. The mobile phase consisted of 0.1 M citrate–phosphate buffer, 0.25 mM 1-octanesulfonic acid sodium salt, 0.1 M EDTA, and 7% acetonitrile (pH = 2.56) (all reagents were purchased from Sigma-Aldrich, St. Louis, MO, USA). The mobile phase flow rate was 1 mL/min. FLD detection was carried out at the excitation wavelength of 285 nm. The emission wavelength was set at 310 nm. Peaks of serotonin were identified by the retention time relative to the standard solutions.

### 4.3. Behavior

The following behavioral tests were performed at P30 using standard methods described earlier: Open Field Test (https://www.openscience.ru/index.php?page=ts&item=001&lang=en&lang=ru&lang=en) (accessed on 22 May 2017); Elevated Plus Maze Test (https://www.openscience.ru/index.php?page=ts&item=002&lang=en&lang=en) (accessed on 22 May 2017); Three-Chambered Social Approach Task [29]; and Resident–Intruder Test [36].

#### 4.3.1. Resident–Intruder Test

Male mice (*n* = 21 for each group) were singly housed for 1 week prior to testing. The cages were not cleaned until the end of the experiment. The male intruders were group-housed (five per cage) and matched with resident mice for approximate age and body weight. The unfamiliar male was introduced to the residential cage and the behavior of the resident mice was monitored during 6 min of exposure to a male intruder. One trial per day was conducted at 8:00 pm. The time between the introduction of the intruder and the first biting attack was considered the attack latency.

#### 4.3.2. Open Field Test

We used equipment adapted for mice (*n* = 12 for each group). It included a round arena with small holes in the floor, divided into three zones—near-wall, intermediate, and central (zones A, B, and C, respectively). Animals were placed in zone A (near-wall) and the test lasted 5 min. We recorded horizontal motor activity, which included running along different trajectories and circling around one place; the speed of movement of the animals and the time without movement; and the duration of stay in each zone of the arena.

#### 4.3.3. Elevated Plus Maze Test

Standard equipment with two open and two closed (dark) arms, adapted for mice (*n* = 12 for each group), was used. It is believed that the choice of closed arms is due to stress, while the open arms are chosen at a lower level of anxiety. Animals were placed in the center of the Elevated Plus Maze on the open arm so that the entrances to the closed arms were in the visible zone. The test time was 5 min. The time spent in closed and open arms, head hanging, and speed of movement of the animals were recorded.

#### 4.3.4. Three-Chambered Social Approach Task

In the Three-Chambered Social Approach Task (*n* = 8 for control group; *n* = 20 for both experimental groups), the number of episodes and duration of the following events were recorded: rears, climbing on the walls of the chamber or the rods of the cylinder, grooming, immobility, entering chambers with the mice in the cylinders, staying in the area near the cylinder, staying in the area far from the cylinder, staying in the chamber without subjects and objects, and mutual sniffing with the mouse in the cylinder. In the case when the animal was able to move freely throughout the entire area, the number of entries and the duration of stay in the area near the first presented cylinder with the mouse and the area far from it and the number and duration of sniffing events with the first mouse and climbing events along the rods of the first cylinder were additionally recorded. All data are presented for every 5 min of observation.

The results of the Open Field and Elevated Plus Maze Tests were processed using special software (“Open Science”, Russia). Animal tracking was performed and standard behavior parameters were processed (characterizing the animal’s speed of movement, trajectory, time spent in different zones, etc.). The results of the Three-Chambered Social Approach Task were processed using the RealTimer program (“Open Science”, Russia).

### 4.4. Statistical Analysis

Statistical analysis was performed using SigmaPlot 12.0 software. In the analyses of statistical significance in serotonin measurements, non-parametric Mann–Whitney tests were applied to compare the groups. Data are presented as the mean ± standard error of mean (SEM).

For the behavior tests, statistical analysis was performed using GraphPad Prism 5 software. To process the results of the Open Field Test, Elevated Plus Maze Test, and Three-Chambered Social Approach Task, the non-parametric Mann–Whitney test was applied to compare the groups. To process the results of the Resident–Intruder Test, contingency tables and Fischer’s exact test were used.

## Data Availability

The raw data supporting the conclusions of this article will be made available by the authors upon request.

## Abbreviations

E, embryonic day; P, day of postnatal development; 5-HTP, 5-hydroxytryptophan; HPLC, high performance liquid chromatography; FLD, fluorescence detector; 5-HT, 5-hydroxytryptamine or serotonin; SERT, serotonin transporter; SSRIs, selective serotonin reuptake inhibitors.
